# Gender and physician specialization and practice settings in Ecuador: a qualitative study

**DOI:** 10.1186/s12913-016-1917-1

**Published:** 2016-11-17

**Authors:** Rita Bedoya-Vaca, Kathryn P. Derose, Natalia Romero-Sandoval

**Affiliations:** 1 Ecuadorian Ministry of Public Health, Vía San José de Minas a 3,5 Km de Perucho, CP. EC170174 Quito, Ecuador; 2 RAND Corporation, 1776 Main St, Santa Monica, CA 90401 USA; 3 Facultad de Ciencias Médicas, de la Salud y de la Vida, Universidad Internacional del Ecuador, Avda. Jorge Fernández s/n y Simón Bolívar, Quito, Ecuador

**Keywords:** Ecuador, Gender, Medical profession, Qualitative

## Abstract

**Background:**

The increasing proportion of women in the medical profession is a worldwide phenomenon often called the “feminization of medicine.” However, it is understudied in low and middle-income countries, particularly in Latin America.

**Methods:**

Using a qualitative, descriptive design, we explored the influence of gender and other factors on physician career decision-making and experiences, including medical specialty and public vs. private practice, in Quito, Ecuador, through in-depth, semi-structured interviews (*n =* 31) in 2014. Theoretical sampling was used to obtain approximately equal numbers of women and men and a range of medical specialties and practice settings; data saturation was used to determine sample size. Transcripts were analyzed using content coding procedures to mark quotations related to major topics and sub-themes included in the interview guide and inductive (grounded theory) approaches to identify new themes and sub-themes.

**Results:**

Gendered norms regarding women’s primary role in childrearing, along with social class or economic resources, strongly influenced physicians’ choice of medical specialty and practice settings. Women physicians, especially surgeons, have had to “pay the price” socially, often remaining single and/or childless, or ending up divorced; in addition, both women and men face limited opportunities for medical residency training in Ecuador, thus specialty is determined by economic resources and “opportunity.” Women physicians often experience discrimination from patients, nurses, and, sometimes, other physicians, which has limited their mobility and ability to operate independently and in the private sector. The public sector, where patients cannot “choose” their doctors, offers women more opportunities for professional success and advancement, and the regular hours enable organizing work and family responsibilities. However, the public sector has generally much less flexibility than the private sector, making it more difficult to balance work and family responsibilities.

**Conclusion:**

Women may outnumber men in medicine in Ecuador and across many parts of the world, but a number of structural issues-economic, social, and cultural-must be addressed for women to establish themselves in a wide variety of medical specialties and practice settings and for countries to realize the benefit of the investments being made to train and employ them.

**Electronic supplementary material:**

The online version of this article (doi:10.1186/s12913-016-1917-1) contains supplementary material, which is available to authorized users.

## Background

The increasing proportion of women in the medical profession is a worldwide phenomenon, often referred to as the “feminization of medicine” [1–3]. Women now comprise a majority or near-majority of medical students and predominate in certain specialties (e.g., pediatrics, obstetrics and gynecology, dermatology, psychiatry) in many high-income countries. For example, in the United States (U.S.), 48% of medical students were women in 2013–2014, up from just 7% in 1965–66 [4] and women now account for more than half of graduate trainees in seven specialties [5]. Similar trends are found across the United Kingdom, Canada, Australia, and various European countries.

Women are also an increasing proportion of the physician workforce in low-and middle-income countries; however, the phenomenon is generally less well studied. In 1994, 50% of medical students completing university medical training in Mexico were women, but an in-depth study of practicing women physicians in this same time frame found a general lack of mobility due to constraining factors such as household gender roles and health institution structures [6]. Furthermore, subsequent studies have found that women physicians in Mexico are underemployed, though the reasons for this (e.g., whether this is because of discrimination in graduate training, occupational choices, or working in public vs. private sectors) are not completely understood [7]. A recent study including physicians in three African capital cities found the proportion of women physicians to range from 28.1% in Bissau, Guinea-Bissau to 56.4% in Praia, Cape Verde, and that women across all three cities were over-represented in the younger age groups [8]. Further, women physicians also predominated in primary care specialties and on average worked fewer hours per week than their male peers, particularly in the private sector.

Ecuador has also experienced an increase in the proportion of women in the medical field; however, few studies on this topic have been conducted. In quantitative analyses, Bedoya Vaca et al. [9] found that between 2008 and 2012, more women than men entered medical school, but fewer women than men graduated from private universities, unlike public universities. Further, during the same period, the Gender Parity Index (ratio of women to men) among physicians working in the public system decreased by 59%, from 2.36 to 1.41, while the number of vacant posts in the public system increased from 1.8 to 11.0% [9]. These trends suggest gender disparities that may present problems for maintaining an adequate supply of physicians, especially in Ecuador’s public sector.

These potential gender disparities raise the broader question of what “feminization” of the medical field really means. Is it merely increasing representation or proportion of women in medicine? Or does it have to do with more equitable opportunities for women to develop careers as physicians? To better understand the “feminization” of the medical profession in Ecuador, we conducted a qualitative study. The primary research questions were: 1) To what extent does gender influence the decision to study medicine and particular medical specialties? 2) To what extent does gender influence professional practice of medicine, including whether to practice in the public or private sectors?

### Theoretical perspectives

As noted by Riska [10], previous studies of gender and medical careers have tended to use one or both of the following explanatory frameworks. The primary framework has been individual and draws on the socialization or sex-role theory (and later iterations): i.e., women and men choose careers that reflect their individual preferences and socio-cultural roles; thus, women physicians tend to choose medical specialties and practice settings and circumstances (e.g., part-time) that enable them to balance work and family. Another framework is more structural and focuses on the social factors that help and hinder women and men in their careers – e.g., role models and barriers such as the so-called “glass ceiling” [11], “leaky pipeline” [12] and “sticky floor” [13]. “Glass ceiling” refers to invisible barriers that keep individuals from obtaining top positions in hierarchical organization; “leaky pipeline” refers to barriers that cause individuals to drop out at various stages of professional development; and “sticky floor” refers to forces that keep certain individuals from progressing up the job ladder. Such barriers for women physicians include hierarchy in academic medicine [14] and manifestations of sexism in the medical environment [15]. Similar barriers exist for women across various labor sectors and are often described as vertical segregation and horizontal segregation [16]. Vertical segregation refers to the lack of women in top management positions (similar to “glass ceiling”), while horizontal segregation refers to the concentration of women in lower paying and lower prestige positions.

Previous studies of the feminization of the medical profession have tended to be quantitative and focus on high income countries. The gendered themes identified through these studies vary somewhat across countries. For example, studies in Norway found that more women than men leave hospital-based specialties such as surgery and internal medicine during training because of work-family role conflict [17, 18]. On the other hand, studies from the Netherlands and Germany have suggested that work-family balance and the possibility of part-time schedules were important to both male and female physicians with young children [19–21]. Another theme identified across various countries is that despite the increasing proportion of women in medicine, their careers progress much more slowly than their male colleagues (e.g., in gaining permanent positions, significant representation in hierarchical medical positions, etc.) [13, 22–24]. A related but slightly different perspective on this theme comes from a study in Scotland, which found that women primary care providers tend to work fewer hours and are less likely to engage in teaching and administrative duties than their male colleagues, suggesting important implications for countries’ physician workforces [25]. However, a systematic review of studies across multiple high income countries found that these differences in hours were due primarily to women’s role in childrearing [2].

Qualitative studies on the feminization of medicine have been more limited in general and, again, focused on high income countries. One gendered theme identified is that women in academic medicine in the U.S. face a number of barriers to career advancement, including the hierarchical structure of academic medicine, constraints of traditional gender roles, sexism from colleagues and superiors, and lack of effective mentors [14, 15]. Another study, from Spain, found that most women physicians did not plan for professional goals and/or intertwined them with family needs, although goal planning and professional development were more common among women who were health care center directors [26]. A study from the U.K. among hospital-based specialists (“consultants”) that used both interviews and observations found a number of gender-related differences-for example, female consultants tended to be less dominating and more nurturing and conversational with patients, but described higher levels of stress regarding work-life balance and gender discrimination from colleagues and lack of support from nursing staff [27].

Further exploration is therefore needed, particularly in low and middle income countries and using qualitative methods, to understand the extent to which gendered cultural norms and structural factors exert their influences on physicians’ careers. As women comprise an increasing proportion of the physician workforce, it is important to gauge the extent to which these roles can be combined with other social roles (e.g., motherhood) as well as what barriers exist to successful careers. Since these can only be fully understood within healthcare system and other contextual factors related to medical careers, we first provide an overview of these factors in Ecuador.

### Medical career trajectories and the healthcare system in Ecuador

Physician’s career trajectories in Ecuador generally begin at 18 years of age, immediately after graduation from high school. Since 2007, medical education at Ecuadorian public universities is free, however, only those who score the highest on the national entrance exam gain admission. Private universities have a different exam, but only those who can afford to pay the cost of private medical education are able to matriculate. Private tuition usually runs about $6000–$12,000 annually, whereas minimum wage in 2016 is $366 per month or just under $5000 per year [28]. Medical school lasts 6 years plus an obligatory 1 year of practicing in a rural (underserved) area; those who complete the 7 years are qualified as general practitioners.

Specialty training is restricted, because the available residency spots in Ecuador are few relative to the number that graduate from public and private universities and some specialties are not offered in country. Those with economic resources or who can obtain funding from the Ecuadorian government can go abroad for specialty training, while those without resources and who are unable to obtain an in-county residency spot must work as a general practitioner. Like in the U.S. and other industrialized countries, specialty training generally lasts 3 years, and subspecialty an additional 1–2 years, after which physicians seek to establish themselves in the public and/or private sectors.

The public and private sectors in Ecuador are two independent health system components, both under control of the Ministry of Public Health (*Ministerio de Salud Públic*a or MSP). Since 2012, when the MSP implemented a law requiring physicians working in the public sector to work at least 8 h each day and 40 h per week (Resolution No. MRL-2011-000033), physicians have essentially had to choose between working in the public or private sectors (prior to this, physicians often elected to do part-time in each). In 2013, a total of 12,519 doctors (47.5% of all doctors) were working in the public system and there were 3,458 ambulatory centers and 765 hospitals [29]. During the same year, the private system had 13,851 doctors (52.5% of all doctors) and more than 10,000 primary care offices and 728 clinics and hospitals.

In contrast to physicians, patients in Ecuador are free to seek care in either system, depending on their personal financial and coverage situations. The public system comprises 4 sub-systems, each with their own facilities and personnel, which serve the following groups: 1) Social Security (all formal sector employees and their families); 2) military; 3) police; and 4) indigent or those not covered in #1–3 and no private coverage. In general, patients seeking care in any of these public sub-systems cannot “choose” their doctors. The private system also has its own facilities and providers, and patients can seek care there if they have private insurance (sometimes provided by employers and other times purchased individually) or can self-pay to cover the fee-for-service costs. Patients seeking care in the private sector can generally choose the doctor they want to see.

Thus, critical decision points of a physician in Ecuador are: 1) whether to pursue medicine (high school or around 16–17 years of age); 2) whether and what to pursue as specialization (around 24–25 years of age); and 3) what setting to practice in, e.g., public vs. private (around 24–25 years of age for general practitioners or 27–32 years of age for specialists and subspecialists). Given that these career decisions are being taken when physicians are also likely to be deciding about family formation as well, we used these decision points as our overall focus of inquiry, bringing a gender lens – i.e., do the factors that affect these decisions appear to be the same or different for women and men?

## Methods

### Collaborative team

The team involved in this research included two Ecuadorian female family medicine physicians, one in clinical practice for the Ministry of Public Health and the other an academic at a local university. Both brought their experiences as women physicians to bear, but from different perspectives (one more immersed in the public health care system and the other with some distance). The other author is a social scientist from the U.S. who lived in Ecuador for 6 years and thus is very familiar with the cultural context, yet brings an outsider perspective.

### Sample and procedure

Using a qualitative, descriptive design, in-depth interviews were conducted from April to July 2014 with medical specialists working in direct patient care in the public and/or private systems (primary, secondary, or tertiary care) in Quito, the capital of Ecuador. “Medical specialists” in Ecuador are those who have completed a residency after graduating from medical school (e.g., family medicine, pediatrics, internal medicine, orthopedic surgery, etc.) – i.e., not those who are general practitioners. Several outreach strategies were used to recruit participants, including: letters from department heads in the public and private systems describing the study, eligibility criteria, and how to get in contact with the principal investigator if interested; suggestions from key informants (one from the public sector and one from the private sector); and snow-ball sampling. Initial contact with potential participants was made via telephone, and, if recruitment was successful, a mutually agreed upon time and place for the interview was set (usually at the physicians’ offices). Theoretical sampling, which is used to build theory and takes place during the collection and analysis of data to build extra heterogeneity into the sample [30], was used to include approximately equal numbers of women and men physicians and across a range of medical specialties and practice settings. Having a range on these dimensions (gender, specialty, and practice setting) was considered important to capturing the range of experiences in the population. The criterion of data saturation [31] was used to determine ultimate sample size (*n =* 31).

Interviews were conducted by the first author using a semi-structured interview guide (see Additional file 1) that was pilot-tested with 4 physicians (2 men and 2 women, 2 from public and 2 from private sector) and subsequently refined based on the pilot experience and feedback from experts in gender and researchers that focus on women and work. Interview topics included: influences on their decisions to study medicine and particular medical specialties, perspectives on gender and medical specialties, gender-related discrimination and problems experienced during training and professional practice, and advantages and disadvantages of working in public and private health sectors. Interviews lasted on average 60 min [range 45 min–75 min] and were audio-recorded (with permission) and transcribed verbatim in Spanish.

### Data analysis

Transcripts were analyzed in Spanish using content coding procedures to mark quotations related to major topics and sub-themes included in the interview guide [32–34] and inductive (grounded theory) approaches to identify new themes and sub-themes [35, 36]. The first author used an inductive approach initially, reading through all transcripts as they became available and noting emergent themes. This use of a grounded theory approach was important because of the overall goals of the study to build theory and understanding about the so-called “feminization of medicine” that are grounded in physicians’ experiences and perspectives. She then created a coding schema using the topics from the interview guide and emergent themes, which was refined by consulting with the other two co-authors. The final coding schema (see Table 1) was then applied to all 31 transcripts using NVivo 10. Coding reports, which provided the universe of the tagged discourse for each subcategory, enabled the researchers to identify the prominent themes across each category, bringing to the analysis a particular focus on the extent to which gender had influenced the trajectories of participating physicians (e.g., whether to study medicine, what specialty to follow, whether to pursue work in the public or private sectors, etc.). However, we also allowed other factors that might have influenced these critical decision points to also emerge as important themes. Once the overall themes were agreed upon by the researchers, exemplary quotations that demonstrated the range of experiences, and in particular similarities and differences among women and men physicians, were selected and translated to English for inclusion in the paper.

**Table 1:** Final coding scheme

Category	Subcategory
1. Concepts about gender and sex	1. Concepts about terms "sex" and "gender" 2. Concepts about feminization of the medical profession
2. Factors that influenced decision to become a physician	1. Wanting to serve (*vocación*) 2. Role models 3. Individual choice 4. Profitable profession
3. Factors that influenced choice of medical specialization	1. Affinity for certain area of medicine 2. Opportunities and availability of economic resources for training 3. Gender and motherhood
4. Factors influencing professional medical practice	1. Discrimination towards women physicians 2. Balancing family and professional responsibilities
5. Advantages and disadvantages of working in the public and private systems	1. Advantages of working in the public system 2. Advantages of working in the private system 3. Disadvantages of working in the public system 4. Disadvantages of working in the private system

## Results

Participant characteristics (total and by gender) are provided in Table 2. Of the 31 participants, just over half (55%) were women. A similar percentage overall was married, however marital status varied by gender, with most men (89%) married and most women (65%) single or divorced. A similar proportion of men’s and women’s parents had been professionals (43 and 41%, respectively), however men interviewed were more likely than women interviewed to have had physician parents (29 and 12%, respectively). In terms of medical training, most (61%) had trained at public medical schools (65% of women and 57% of men). However, current practice setting varied dramatically, with most of the men interviewed (79%) practicing in the private sector and most of the women (70%) practicing in the public sector.

**Table 2:** Participant characteristics

Characteristic	Total (*n =* 31)	Women (*n =* 17)	Men (*n =* 14)
Median age [range] in years	39 [35–45]	38 [35–45]	39 [36–45]
Marital status			
Married	17 (55%)	6 (35%)	11 (79%)
Single	8 (26%)	8 (47%)	0
Divorced	4 (13%)	3 (18%)	1 (7%)
Live with partner (unión libre)	2 (6%)	0	2 (14%)
Parents’ occupational status			
Physician	6 (19%)	2 (12%)	4 (29%)
Other professional	7 (23%)	5 (29%)	2 (14%)
Non-professional	18 (58%)	10 (59%)	8 (57%)
Type of medical school			
Public	19 (61%)	11 (65%)	8 (57%)
Private	12 (39%)	6 (35%)	6 (43%)
Current practice setting			
Public	15 (48%)	12 (71%)	3 (21%)
Private	16 (52%)	5 (29%)	11 (79%)

### Factors influencing the decision to pursue medicine as a career

Analysis of the sub-themes from this category identified several main reasons that men and women decided to become physicians, although these reasons did not seem to be influenced by gender. First, respondents talked about having been motivated to study medicine by a desire to serve (*vocación)*, often because of their own family members’ experience with illness and/or because of the strong cultural influence of religion. Second, women and men interviewed described how their decisions to study medicine were motivated in part by admiration and gratitude towards physicians who had played important roles in their lives, such as a close family friend or their parents. Third, there were some physicians, both women and men, who expressed a strong self-motivation to become a physician, even when met with opposition from parents, who felt that other professions were preferable. Finally, women and men interviewed also indicated that seeing medicine as profitable was an additional motivating factor, often reinforced by parents and other extended family members.

### Factors influencing choice of medical specialty

Although we did not find strong evidence of gender having influenced decisions to pursue medicine, we did find several ways that it appeared to have influenced choice of medical specialty. Analyses of themes regarding this issue identified three subcategories: affinity for certain specialties, (economic) opportunity, and, for women physicians, motherhood.

#### Affinity

Some physicians interviewed expressed that a clear preference or affinity for a certain specialty had influenced their decisions. However, this seemed to be more related to economic resources, since this view was most often expressed by physicians who had resources to go abroad for their residencies, rather than gender. The one case where gender did seem to play a role was a woman ob-gyn working in a private hospital who said: “Being a woman influenced me greatly because when I decided on my specialty. I was doing my rural year, and, theoretically, I was going to be a pediatrician, but I realized that I felt better treating women, so I found a postgraduate program in Mexico.”

#### “Opportunities” and socio-economic resources

More consistent influences on selection of medical specialty were the “opportunities” available for post-graduate training. Those who did not have economic resources to go abroad for their residencies had to choose a specialty with ample in-country spots or where they had a particular connection. Sometimes these “opportunities” appeared to be intertwined with gender-specific roles. For example, a male physician working in the private sector shared how he ended up doing emergency medicine due to lack of economic resources to go abroad and the fact that he already had a family and thus needed to fulfill the traditional role of provider:

What’s more, I never thought of being an emergency medicine physician, within my range of possibilities was to be a pediatrician or neurologist, or something like that, you know? What happened was that my direct boss in the clinic where I worked was named director of the postgraduate program in emergency medicine [in Ecuador], so he gave me the opportunity, because I couldn’t go abroad to study what I wanted because I didn’t have the money and I had a family, so I studied what I had the opportunity for.

Another potentially gendered example of this was a female physician working in the private sector who was the eldest of several siblings shared how the death of her father (and loss of economic support) influenced her choice of specialty, as she felt compelled to fulfill the cultural expectations of a woman giving up her career expectations to take care of her family:

My intention was to be a gastroenterologist or internal medicine, but at the end of my studies, my father passed away, and he was my economic support. We had planned that I could go to Mexico for my specialization, but when he died right when I was doing my internship, I was left in charge of the family. My siblings were in school and my mother never worked, the only one working was me, so I did the postgraduate program [in Ecuador] that I had the chance to do.

#### Gender – motherhood

Men and women interviewed felt that there are medical specialties that are more appropriate for men and those that are more appropriate for women, and underlying these views was a strong cultural view about the importance of mothers. Although physicians interviewed recognized that men and women are equally capable of studying any specialty, they considered surgical specialties that require night shifts and are more demanding as difficult to reconcile with motherhood. A male primary care provider in the public sector shared how men were better matched for certain specialties and emphasized the importance of the mother’s role in the home:

I think there are specialties that could be better suited for men, for example trauma, where strength is needed and the same for intensive care and having to be on-call constantly. I think the presence of the mother in the home is fundamental. So leaving the home to do these other things, I believe, creates some difficulty.

Women working in the public sector agreed with men’s belief that there are specialties more appropriate for men and others more appropriate for women, with those for women being ones that allow combining family life with professional life. For example, one woman described how her non-physician husband admonished her to select a specialty that would not cause her to neglect her family:

My husband is not a doctor, but he told me when I was choosing my specialization, that I choose a specialization in which I wouldn’t have to leave them at night, so I wouldn’t neglect my daughter. Yes, yes that had a lot of influence. So I went a bit blindly to the postgraduate school, to find out which programs didn’t involve working overnight.

Nevertheless, there were some women in the public sector who opted for specialties considered “men’s work,” however, these tended to also be women who were single and childless. For example, one woman explains how she had to abandon her dream of a family to pursue orthopedics and, although she is happy with her decision, she notes the double standard:

To begin with, being a woman and having a profession is a little complicated, as I said at the start, I’m single. I chose a profession and left behind the dream of a family and of being a woman and decided to study, to research. Orthopedics is a demanding specialty where one needs to be available 24 hours a day. This makes it impossible for women to reconcile orthopedics with family life. That’s why I decided on my specialization, and I’m happy with my decision. Still, I think it’s unfair because they [men] do have families.

Another woman shared how she never even considered getting married or having children and attributes her success in her field and chief of staff at a private hospital *as a woman* to this fact:

I never thought of getting married, much less in having children. I was completely convinced that women who were doctors could not choose to be mothers or wives. I believed that you had to do one or the other. I’ve fulfilled that, and I feel in an equal situation to a man without limitations, maybe that’s why it hasn’t been hard for me to be chief of staff at this hospital.

Similarly, a woman surgeon in the public sector described how motherhood is incompatible with success as a surgeon:

When a woman decides to be a surgeon or anything else, being a woman and young will give her problems. So one has to go in knowing that, at some point, you will have to fight for your place. At least that’s what happened to me with my colleagues. To be able to learn, to be able to do, to be able to be, I had to be better than my [male] colleagues, that’s the only way I earned respect. But of course, in these circumstances, if one decides to be a mother, it’s much more complicated.

In addition to the difficulties of managing being a mother and a physician (particularly for women in surgical and other demanding specialties), women physicians described challenges in gaining the respect and acceptance of patients. Several men interviewed confirmed these challenges, stating that although professors and male colleagues treated women and physicians equally, oftentimes it was the patients who did not see women as capable or even as physicians. For example, one man working in a public hospital explained how during residency training, he observed this discrimination from patients towards women residents, including the chief resident:

When I was doing my postgraduate studies there were a lot of women, they worked the same as the men, it was the patients that looked for the man to be making decisions in critical cases. For example they said to my female colleague, and I’m talking about the chief resident, “Wouldn’t it be better if you called the male doctor so that he can decide what to do?” So my colleague got mad and said, “Here I’m the boss of all these doctors, so I’m the one who makes the decisions!” Many times women need to yell and have an aggressive attitude in order for the patients to respect them.

In contrast, one man from the private sector described situations in his medical training where professors seemed to favor women, for example, granting them more time off:

It seems to me that there was a bit of discrimination. We were a mixed group; sometimes the women received, from some of the doctors, the professors, a more favorable treatment, they were given privileges, I don’t know if more opportunities but privileges in that moment. I mean, more time off, permission to leave early, I mean, a little more consideration.

### Factors influencing professional medical practice

Analyses of themes regarding men and women’s experiences in professional medical practice, including whether to work in public or private settings, identified two primary themes: discrimination against women physicians, and balancing family and profession.

#### Discrimination towards women physicians

Women physicians, particularly those in surgical specialties, described many structural barriers to exercising their profession, especially in the private sector but also in the public sector. Discrimination towards women physicians was described at length, and came from various sources, including physician colleagues, other women in the healthcare workforce (e.g., nurses), and patients.

In terms of colleagues/bosses, a common complaint was that women surgeons were not included in the operating room schedule. One woman working in a public hospital, who had been trained to do a highly specialized surgical procedure, explained how her chief restricted her use of the operating room:

The issue is not whether you are capable or not, the problem is that the structure is sexist, so creating spaces in operating rooms for women has not been easy, it has been hard work. When I proved to be very skillful and had a specific dexterity for [doing this surgical procedure], my colleagues wouldn’t allow me to have time in the operating room. My boss said I could operate with him, not alone, and I had to apply pressure at other levels for them to assign me time in the operating room.

One woman working in the public sector was warned by the auditor of the private hospital where she had once worked that she would face difficulty practicing there as a woman:

The auditor told me: “Well, doctor, you have two strikes against you, you’re a woman and you’re young. Prepare yourself.” It was very hard, very hard, in a private hospital that was completely sexist, very hard, very hard. But I was there for 3 years and finally in that hospital, it was a lot of work, but I believe that I gained respect.

Another woman working in a private hospital observed that although there were many very good women surgeons, few had had the opportunity to be chief.

Some women in the private sector described discrimination from other women who work with them, such as nurses not carrying out their orders, for example:

More so we had problems with the female nurses that wouldn’t follow our orders and a lot of the time we had to do work that corresponded to them. I noticed that they were always more willing to what our male colleagues asked. There was like a flirtatiousness among them.

Similar to narratives regarding women’s experiences in medical specialty training, another common theme affecting professional practice was discrimination by patients, especially towards women surgeons. For example, this woman from the public sector described how patients also prefer a male surgeon, and how this makes succeeding in the private sector very difficult:

There will always be a preference for men. Patients have much more confidence in male doctors. They still treat us like young women. They don’t accept women physicians. When I was a postgraduate and I had to do a caesarian, and it was very disappointing because the husbands always wanted a man to operate. I thought that in private practice it was going to be very difficult to develop my specialty. The problem isn’t discrimination from professors or colleagues, it’s from patients.

Another woman surgeon from a public hospital surmised that patients don’t feel “secure” with a woman surgeon, and identified clear differences from women surgeons in the public vs. private sectors:

Being a man or a woman influences in a definitive way. I’m a very capable surgeon, and I say this because my colleagues recognize this, but the patients don’t feel safe with a female surgeon. When I was working in a public hospital to pay back my scholarship, the patients never had the option to choose the surgeon. So I would operate on them, and they were very grateful. But when they were going to pay for surgery in a private clinic, the patients wanted to be operated on by a male surgeon. When I married a male surgeon, the patients wanted to be operated on by my husband. For years, I operated with him and I did complicated things, but in the patient’s view, my husband was the surgeon. That frustrated me so much that I ended up getting a divorce.

This discrimination that women physicians face ultimately has an emotional toll, as evidenced by the woman surgeon’s comment that this ultimately led her to divorce. A male emergency physician in the private sector also noted that his discrimination placed additional stress on women physicians, on top of having to deal with patients medical problems:

What I have lived as an emergency physician is that when women are at the top, I mean chiefs of staff, they can make decisions with a cold head, but it’s the patients that disqualify them. And, over time, [these women chiefs] fatigue more quickly because they are not only dealing with medical problems of the patients but also have to sustain their role with an imposing attitude, because, if they don’t, the patients and nurses don’t respect them. So they have to yell more during their shifts.

#### Balancing family and professional responsibilities

Women and men interviewed both talked about the difficulties they encounter in balancing having a family and also being a successful physician. However, they also recognized that the challenges were greater for women physicians, given their culturally determined roles as primary caregiver. One man working in a public hospital described how many of his female colleagues have gotten separated and either do not have kids or postpone motherhood: “Of the women I know here, there a quite a few who have separated from their husbands because they decided to develop themselves professionally and obviously not have children or at least postpone motherhood, and they have ended up separating.” A female primary care provider in the public sector described her mothering role as primary reason for leaving her university post: “Well, you’re doing this interview right in a moment when I’m in a maternal crisis, at this moment I’m deciding to leave the faculty, because I don’t want to leave my son alone. I think that I’ll work for only 8 hours [a day], no more.”

A female primary care provider in the public sector talked about how many of her friends had pursued their careers but later decided that they wanted a child and had to resort to fertility treatments, often abroad:

I have a lot of [female physician] friends who decided not to marry or preferred being physicians instead of mothers. And when they achieved that, when they fulfilled their goals, they decided to then have a child and it was late. I have three or four friends that were in fertility treatment, including abroad. The majority of us that have children, we had them in university when we were very young. The other group are single or don’t have children.

Women who do decide to pursue motherhood and medicine, but given the strong cultural beliefs about what it means to be a mother, gaining an optimal balance remains challenging and can influence women’s mental health and sense of well-being. One male primary care provider in the private sector expressed these cultural beliefs, indicating that children are best raised by their mothers rather than a nanny.

It’s important to establish that there is a point where, biologically, in order for a child to grow healthily, they need the protective figure of a mother and father, indispensably a maternal figure. And that requires one of the two to sacrifice a part of their professional life, a part, it doesn’t have to be all or nothing. There will never be good mothering from a nanny; they can play a marvelous role, but it will never be the same.

Physicians interviewed did not think that working part-time was acceptable professionally, and part-time work and part-time stay at home mom can create internal conflict for women, as noted by another male primary care provider in the private sector (who is also a psychotherapist):

When women opt to work part-time, they’re not taken seriously, they’re not valued the same way. So they feel like they’re not doing anything well, they’re not full-time at home or at work, so they live a constant conflict. As a psychotherapist, I have treated many colleagues with these conflicts, and sometimes it can give them serious behavioral difficulties.

In contrast, a man working in a private hospital suggested that changes may be on the horizon and perhaps women physicians in the future will not face this dichotomy of medical profession or motherhood:

I see more possibilities for women, in the sense that the new generation of men continue to become more involved in domestic work....and probably the next generations are in process, but for the female doctor that has children and a husband, it seems to me very difficult to arrive home and continue to work.

## Discussion

Our qualitative study found strong evidence that both the socialization or sex role theory *and* structural barriers influence men and women physicians’ career decisions in Ecuador.

In particular, we found that gendered norms (socialization) and individuals’ social class or economic resources (structural factors) strongly influenced physicians’ choice of medical specialty. The idea that women’s first priority should be family and childrearing remains normative in Ecuador, and this cultural view constrained women’s choices, as both they and their male colleagues conceded that certain specialties were difficult to reconcile with motherhood. Women who opted for specialties considered “men’s work” (orthopedics, intensive care, surgical specialties) have had to “pay the price” socially, often remaining single and/or childless, or ending up divorced, similar to what has been found among women academics more generally in English-speaking countries [37, 38]. However, in addition to the influence of gendered norms, women and men in Ecuador face another type of structural barrier that influences medical specialty-limited opportunities for post-graduate specialty training (residency) in Ecuador. This reality means that social class and available economic resources determine whether a physician can pursue their preferred specialty, which often requires spending 3–5 years abroad, or, if they must, choosing a specialty based on the “opportunities” are presented to them.

We also found that gendered norms influencing choice of specialty interacted both with social class or economic resources and other structural factors to influence women physician’s choices for professional practice (public vs. private). For example, in addition to a social cost, women physicians often have to pay a price professionally, facing discrimination from patients, nurses, and, in some cases, other physicians, and these structural barriers have limited their mobility and ability to operate independently and in the private sector. Accordingly, women in surgical (“male”) specialties often find that the public sector, where patients cannot “choose” their doctors, offers these women more opportunities for professional success and advancement – i.e., less of a sticky floor and glass ceiling. Further, following the socialization theory, women physicians wanting to organize their work and family lives found the regular hours of the public sector appealing. Thus, it is not surprising that a majority of women interviewed (71%) were working in the public sector, whereas only 21% of men were. However, working in the public sector has generally much less flexibility than the private sector, in terms of being able to coordinate with children’s school activities or emergencies, making it more difficult to balance work and family life. Similarly, women physicians in the SwissMedCareer study preferred to work in private practice settings for this same reason [23]. But in Ecuador, women physicians face a tradeoff: the private sector offers them some more flexibility with work-family balance but is also where they face discrimination from patients who prefer a male physician.

Ecuador thus faces challenges, as do many other countries, in managing this transition to an increasingly majority female physician workforce. Even in countries such as Norway, where progressive social policies in the 1980s and 1990s have enabled women to combine employment and family/children, and in spite of medical career opportunities being formally equal, studies have found that women physicians’ careers are more affected by family responsibilities than those of male physicians’ [18] and that certain specialties such as surgery and internal medicine make it difficult for women to combine work and family [17]. Thus, further efforts are needed to develop support systems for women physicians who want to also have families – and increasingly this is important for men as well [19, 21]. High quality and affordable childcare facilities, a more equal division of domestic and caring responsibilities, and the ability to adjust work to family responsibilities when having small children are innovations that have been developed elsewhere [18, 23].

In addition, it is important to consider ways to enhance women’s career advancement within academic medicine and other hierarchical medical structures. Studies across diverse settings such as Japan, Scandinavia, Russia, Spain and other parts of Europe, and the U.S. have found that even when women represent a high proportion of physician workforce, they may continue to be under-represented in positions of leadership and prestige – all aspects of the “leaky pipeline,” “glass ceiling,” and vertical segregation phenomena [12, 22, 24, 39]. In Ecuador, the first to fall through the cracks are women with children and few economic resources, as they face the largest number of barriers to complete a medical specialty, since residency spots are restricted in Ecuador and only physicians with economic resources and usually without children can go abroad to study.

This intersection of gender and class issues was an important underlying theme in our findings, yet rarely is this discussed in the literature on feminization of the medical profession. As noted earlier, since 2007, medical education at Ecuadorian public universities is free to those able to gain entrance (though obtaining the highest scores on the national entrance exam), thus enabling individuals from low socio-economic backgrounds to enter this higher socio-economic profession. However, studies in other countries such as Australia have found that medical school applicants with low socioeconomic backgrounds are more adversely impacted by the use of cognitive ability testing (while interviews and actual performance in medical school are not affected), particularly if they are women [40]. Thus gender and class can intersect to affect who gets into medical school. Further, Gjerberg [17] notes the intersection of gender and class in that female specialists in surgical and other male dominated specialties more often have higher social status than women in other specialties, possibly needing to “compensate” for being of the “wrong” gender. In Ecuador, there are likely additional, more practical ways that gender and class intersect to influence specialty, since women of higher social class have more opportunities for “choosing” their residencies or specialties and having high quality childcare that enables them to engage in surgical and other hospital-based work. Therefore, it is important to consider how the intersectionality of gender and class contribute to the distribution of women in various medical specialties (horizontal segregation), and the various “leaks” in the physician career pipeline in Ecuador and elsewhere.

Further, these “leaks” that women face in the career pipeline persist beyond residency training, into practice settings and academic appointments and may help explain the earlier quantitative figures noted [9] that suggested that women in Ecuador were leaving the medical field, either during medical school or, potentially, while practicing (or at least leaving the public sector). Our qualitative data suggest that these decisions are not taken lightly, nor are they due only to women’s primary role in childrearing. Instead, the broader patriarchal attitudes that persist among some colleagues and patients constrained women physicians’ abilities to practice, particularly in specialties considered “men’s domain” and within the private sector. These findings support calls for more concerted efforts to address gender discrimination and inequality in the health workforce globally [41].

This variation across specialties raises an important limitation of our study. Although we interviewed men and women physicians across a range of levels of care (primary, secondary, and tertiary) and public and private settings, we did not interview a male and female physician for each specialty. Additional studies that systematically include men and women from various specialties may be needed to identify the full range of experiences within each specialty. Another limitation is that we did not interview those who “leaked” out-i.e., the women who left medical school or dropped out of practice, whose perspectives would have helped us better understand the reasons behind this phenomenon. Finally, although the physicians in our study perceived a clear bias or preference on the part of patients for male surgeons, we did not have data directly from patients to corroborate nor explore this preference. Still, it was clear that patients did play a role in physician’s gendered experiences, particularly in the private sector, and thus studies that include physician and patient perspectives of these experiences could be very enlightening.

## Conclusion

Women may outnumber men in medicine in Ecuador and across many parts of the world, but it is clear that the profession is far from being “feminized,” if one perceives by this a transformation of the field towards more equal opportunities for women to develop careers as physicians. A number of issues-economic, social, and cultural-must be addressed for women to be able to establish themselves in a wide variety of medical specialties and practice settings. Otherwise, the leaks will persist, ultimately proving costly not only to the women who follow medicine as a career path, but also to the countries that sponsor and employ them.

## Additional file

Additional file 1: Semi-structured Interview Guide. (DOCX 15 kb)
