# Supplementary material for: Gender and physician specialization and practice settings in Ecuador: a qualitative study
Source: BMC Health Serv Res. 2016 Nov 17;16:662. doi: 10.1186/s12913-016-1917-1 (PMC5114743; doi:10.1186/s12913-016-1917-1)
Supplement: Additional file 1: — Semi-structured Interview Guide. (DOCX 15 kb) [file 12913_2016_1917_MOESM1_ESM.docx]

Gender and the Medical Profession in Ecuador

**Semi-structured Interview Guide**

1. Opener questions asking about respondents’ understanding or definition of the terms: sex, gender, and feminization.
2. What were the primary motivations behind your decision to pursue medicine as a career? Were these your own decisions or were you advised to do so by others? By whom?
3. What were the primary motivations behind your selection of medical specialty? Did you consider your gender when you had to select your specialty? Do you think that there are specialties more appropriate for men and specialties more appropriate for women?
4. Did you experience gender discrimination in your training?
5. Have you had any problems in professional practice that could be related to gender?
6. What do you think are the advantages of working in the public healthcare system?
7. What do you think are the disadvantages of working the public healthcare system?
8. What do you think are the advantages of working in the private healthcare system?
9. What do you think are the disadvantages of working in the private healthcare system?
10. What time do you start your work day and what does a typical day look like?
11. Are you able to do other types of activities, such as social activities, political activities, sports, etc.?
12. What do you think would be an ideal work schedule for you?
